# Computed Tomography and Cardiac Magnetic Resonance in Ischemic Heart Disease

**DOI:** 10.1016/j.jacc.2016.08.047

**Published:** 2016-11-15

**Authors:** Marc R. Dweck, Michelle C. Williams, Alastair J. Moss, David E. Newby, Zahi A. Fayad

**Affiliations:** a Translational and Molecular Imaging Institute, Icahn School of Medicine at Mount Sinai, New York, New York; b Zena and Michael A. Wiener Cardiovascular Institute, Icahn School of Medicine at Mount Sinai, New York, New York; c Centre for Cardiovascular Science, University of Edinburgh, Edinburgh, United Kingdom

**Keywords:** acute coronary syndrome, atherosclerosis, calcium, coronary artery disease, myocardial infarction, noninvasive imaging

## Abstract

Ischemic heart disease is a complex disease process caused by the development of coronary atherosclerosis, with downstream effects on the left ventricular myocardium. It is characterized by a long preclinical phase, abrupt development of myocardial infarction, and more chronic disease states such as stable angina and ischemic cardiomyopathy. Recent advances in computed tomography (CT) and cardiac magnetic resonance (CMR) now allow detailed imaging of each of these different phases of the disease, potentially allowing ischemic heart disease to be tracked during a patient’s lifetime. In particular, CT has emerged as the noninvasive modality of choice for imaging the coronary arteries, whereas CMR offers detailed assessments of myocardial perfusion, viability, and function. The clinical utility of these techniques is increasingly being supported by robust randomized controlled trial data, although the widespread adoption of cardiac CT and CMR will require further evidence of clinical efficacy and cost effectiveness.

## Central Illustration

**Figure:**
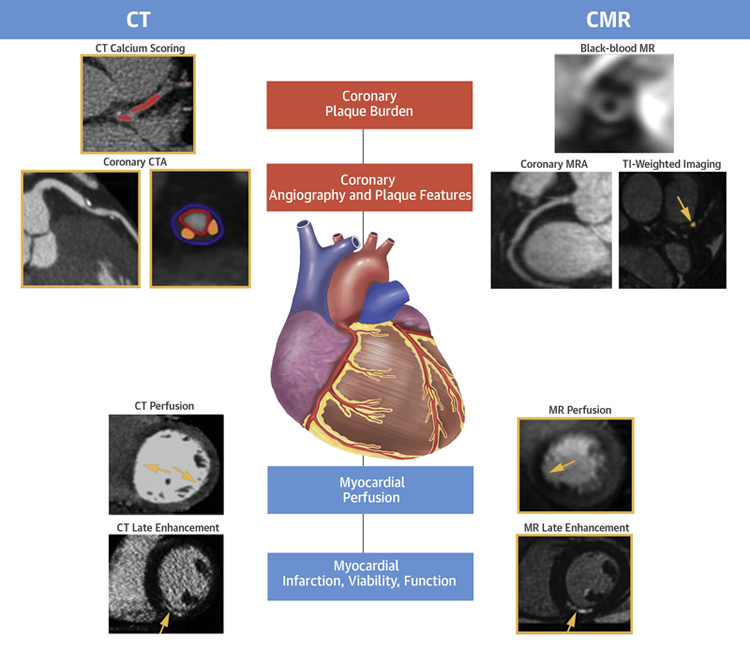

Ischemic heart disease is a complex chronic disease characterized by pathological changes in both the coronary arteries and the myocardium, and which incorporates multiple different phases and clinical syndromes. Modern noninvasive imaging with computed tomography (CT) and cardiac magnetic resonance (CMR) now has the ability to monitor each of these different stages. In particular, CT allows precise imaging of coronary atherosclerosis (plaque burden, angiography, adverse plaque characteristics), whereas CMR allows detailed investigation of the left ventricle (perfusion, infarct visualization, function), although this distinction is increasingly becoming blurred with technological advances. This paper provides a comprehensive review of CT and CMR imaging in ischemic heart disease and compares their relative merits and limitations. Discussion examines their current clinical application and future potential developments, as well as the substantial barriers that exist to widespread adoption.

## Pathophysiology of Ischemic Heart Disease

Coronary atherosclerosis is a chronic progressive disease with a long and mostly unrecognized preclinical phase. The first pathological abnormality, the fatty streak, can be observed as early as the second decade of life (1). Ultimately, these streaks develop into mature atherosclerotic plaques consisting of a central lipid core covered by a fibrous cap. As the plaque grows, the affected vessel expands in an outward direction, preserving both the luminal diameter and blood flow, in a process known as positive remodeling. Consequently, even large plaques can be accommodated without producing symptoms and without being identified on invasive angiography or stress testing. Eventually, the plaque begins to grow into the lumen of the vessel, obstructing blood flow and causing myocardial ischemia and symptoms of angina pectoris. Importantly, although the degree of luminal stenosis is closely related to the development of myocardial ischemia, multiple other factors such as entrance effects, friction, and turbulence, also contribute to increased flow resistance across a particular stenosis (2). Moreover, it is now well established that most myocardial infarctions (MIs) arise from plaques that are nonobstructive on antecedent angiography, in part related to the much greater prevalence of these lesions 3, 4.

Atherosclerotic plaques can remain quiescent for years; indeed, most will remain subclinical during a patient’s lifetime. However, individual plaques can have a major clinical impact when their surface becomes disrupted, initiating thrombus formation and, potentially, vessel occlusion and acute coronary syndromes (ACS). Most commonly, ACS is triggered by acute fibrous cap rupture, which exposes the thrombogenic, tissue factor-rich lipid core to circulating blood. Alternatively, plaque erosion of the endothelium overlying the fibrous cap can lead to the formation of a platelet-rich thrombus, accounting for up to 30% of MIs (5). However, ACS is not an inevitable consequence of fibrous cap disruption. Indeed, subclinical plaque rupture appears common, with up to 70% of obstructive coronary plaques containing histological evidence of previous rupture and subsequent repair (6). The magnitude of the thrombotic response to plaque disruption is therefore also important and is governed by multiple factors, including the thrombogenicity of the blood, flow along the vessel, and constituents of the plaque.

Plaques that rupture and precipitate acute thrombotic events frequently have certain key characteristics on histological examination, including a thin fibrous cap (<65 μm), positive remodeling, a large necrotic core, inflammation, microcalcification, angiogenesis, and plaque hemorrhage. Each of these adverse plaque characteristics, therefore, represents a potential imaging target for identifying plaques at high risk of rupture. Although prospective data have suggested that most of these so-called vulnerable plaques either heal or rupture subclinically rather than cause ACS (7), such features rarely exist in isolation and can serve as a marker of patients with advanced and metabolically active atheroma 7, 8. Invasive imaging studies have demonstrated the presence of multiple high-risk plaques across the coronary vasculature in patients with ACS (9), and postmortem studies have demonstrated that multiple coronary thrombotic events are present at the time of fatal MIs (10). Both of these observations support a pancoronary vulnerability to atherosclerotic plaque rupture. On this basis, interest persists in detecting vulnerable plaque, not because these lesions will necessarily rupture themselves (11), but rather as a means of identifying vulnerable patients, those subjects with active atheroma and a propensity to develop multiple high-risk plaques over time, one of which may ultimately cause an event.

Both myocardial ischemia and infarction can have a profound effect on the structure and function of the left ventricular myocardium. MI results in tissue necrosis and ultimately irreversible areas of scarring, reducing the ability of the heart to both contract and relax. MI also results in areas of stunned myocardium, regions of hypokinesia that are damaged but not infarcted, with the potential to regain function. Similarly, severe myocardial ischemia can result in hibernating myocardium, areas with impaired function that can be restored if blood flow is improved. Ultimately, these insults to cardiac function can lead to congestive cardiac failure and development of ischemic cardiomyopathy.

## Imaging with CT and CMR

The successful application of CT and CMR imaging to the heart was delayed compared with application to static organ systems, principally due to the heart’s complex motion during both the cardiac and the respiratory cycles. However, advances in scanner technology now offer robust methods for motion correction and much improved spatial and temporal resolution, heralding a new era of noninvasive cardiac imaging. Each technology has different strengths and weaknesses (Table 1) that can potentially provide complementary information regarding ischemic heart disease. We focus here on the ability of these technologies to image plaque burden, high-risk plaque characteristics, and luminal stenoses in the coronary arteries, whereas in the left ventricle, we concentrate on the imaging of myocardial perfusion, infarction, viability, and function (Central Illustration).

**Table 1:** Technical Comparison of CT and CMR Approaches to Coronary Angiography

Parameters∗	CT	CMR
Scan acquisition		
Scan duration	0.5–10 s	10–20 min
Spatial resolution	Submillimeter	Millimeter
Temporal resolution	280–420 ms (as good as 65 ms with dual source CT)	<60 ms
Radiation exposure	1–10 mSv (depending on protocol)	No radiation exposure
Advantages	Shorter total scan time • Favored by patients • Increased number of scans per day • Single breath hold Better spatial resolution	Radiation-free imaging • Serial scans • Young patients • Early disease Soft tissue characterization
Limitations	Requirement for adequate rate control Contrast medium reactions (iodine) Contrast induced nephropathy Calcium-related artifacts (the combination of partial volume averaging and photon starvation† makes calcium appear larger on CT) Radiation exposure	Claustrophobia Contrast medium reactions (gadolinium) Risk of nephrogenic systemic fibrosis if glomerular filtration rate <30 ml/min/1.73 m^2^ Metallic implants (including pacemakers and automated implantable cardioverter-defibrillators) Long scan times and limited spatial resolution

CMR = cardiac magnetic resonance; CT = computed tomography.

∗ Imaging parameters depend on the sequence performed. Typical values for coronary angiography are provided for comparison.

† Photon starvation refers to a common artifact seen in the imaging of high attenuation structures, such as calcium.

**Central Illustration:**
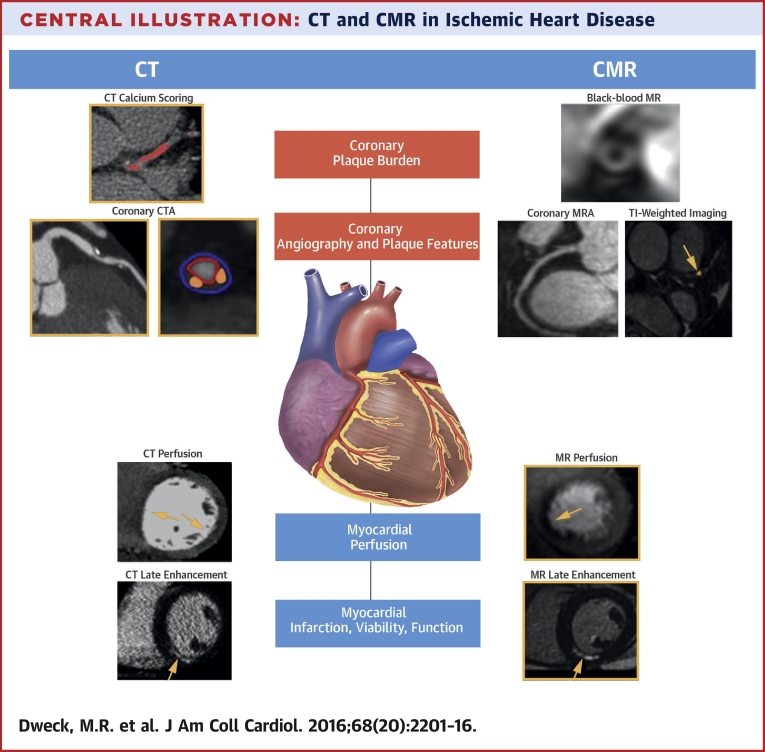
CT and CMR in Ischemic Heart Disease Both CT and CMR offer detailed and comprehensive imaging of ischemic heart disease. Gold-outlined boxes highlight preferred modalities. CT currently holds the advantage for coronary imaging, whereas CMR offers more detailed assessments of the myocardium. **Orange areas** on the coronary CTA outline areas of low attenuation (<30 Hounsfield units) plaque. **Orange arrow** on T1 weighted images indicates an area of high intensity plaque in the coronary arteries. **Orange arrows** on the perfusion images indicate areas of reduced myocardial perfusion during stress. **Orange arrows** on the late enhancement images indicate areas of subendocardial late enhancement consistent with previous myocardial infarction. CMR = cardiac magnetic resonance; CT = computed tomography; MRA = magnetic resonance angiography; MRI = magnetic resonance imaging.

## Coronary Plaque Burden

Coronary plaque burden assessments are useful in identifying the subclinical phase of the disease and providing powerful prediction of adverse cardiovascular events. Although most patients identified on imaging as having coronary atherosclerotic plaque do not subsequently suffer adverse events, the more plaques a subject has, the higher their risk, presumably because this increases the chances of 1 plaque becoming disrupted and causing an event. Imaging plaque burden is, therefore, potentially attractive in terms of both population screening and risk stratification of asymptomatic patients.

**Figure 1:**
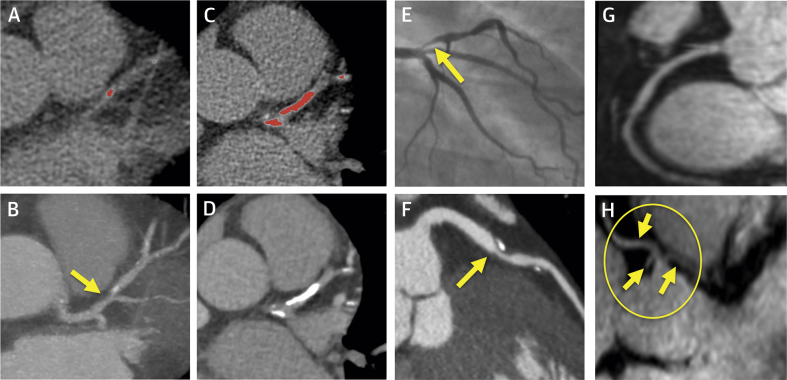
Assessments of Plaque Burden and Coronary Angiography Coronary CTA allows assessment of both luminal stenosis and plaque constituents, calcific and noncalcific. Patient #1 shows matched CT calcium scoring (**orange areas** indicate calcium) **(A)** and coronary CTA images **(B)**. Patient #2 shows matched CT calcium scoring **(C)** and coronary CTA images **(D)**. Patient #3 shows invasive coronary angiogram **(E)** and coronary CTA **(F)**, demonstrating a stenosis in the proximal left anterior descending artery. Coronary MRA provides luminal assessments of the proximal right coronary artery **(G)** and demonstrates aberrant origins of both the left anterior descending and the circumflex arteries from the right coronary sinus **(arrows) (H)**. CTA = computed tomography angiography; other abbreviations as in Central Illustration.

Coronary artery calcium (CAC) scoring uses noncontrast electrocardiographic (ECG)-gated CT to provide accurate and simple measurements of the coronary atherosclerotic burden (Figure 1). In particular, CAC quantifies macroscopic calcium within these vessels by using the Agatston score (12). Coronary macrocalcification is highly specific for atherosclerosis but is usually associated with stable plaques at low risk of rupture. This is supported by autopsy and imaging studies demonstrating that stable plaques are associated with advanced macroscopic calcification, whereas ruptured coronary plaques tend to be associated with the very early stages of micro- or spotty calcification (often with extensive calcification elsewhere in the coronary vasculature) 13, 14, 15. Similarly, other CT studies have demonstrated that more dense coronary calcification is associated with a lower risk of cardiovascular events than less dense calcium (16). Nevertheless, CT calcium scoring provides powerful prognostic information because it provides a surrogate for the total plaque burden that will correlate with the number of unstable, adjacent plaques. A CAC score >300 Agatston units (AU) is associated with a 4-fold higher risk of cardiovascular events than a CAC score of zero (17), which is itself associated with an excellent prognosis. Indeed, “the power of zero” is so good that it can provide a <1% annual mortality rate for up to 15 years in asymptomatic and otherwise low- or intermediate-risk patients (18), justifying the downscaling of preventive treatments (19). High calcium scores are also of use, with several population-based studies demonstrating that addition of calcium scoring to Framingham risk scores 20, 21 improves the prediction of coronary events. On that basis, the 2013 American College of Cardiology/American Heart Association Guideline on the Management of High Cholesterol (22) recommended that a CAC score of >300 AU be used as a modifier to justify statin therapy for primary prevention in adults between 40 and 75 years of age without diabetes and with low-density lipoprotein cholesterol 70 to 189 mg/dl. Although the radiation dose associated with CT calcium scoring is small (2 to 3 mSv), it does remain of concern in the context of multiple repeat assessments or widespread population-based screening programs. Additionally, CT calcium scoring does not quantify the burden of noncalcified plaque, leading to interest in the use of contrast CT imaging. This allows measurement of the total coronary plaque burden, which also provides important prognostic information, refining the prediction of cardiac death and recurrent MI in intermediate-risk patients (23).

CMR black-blood imaging exploits differences in magnetic relaxation properties to generate soft tissue contrast and to differentiate atherosclerotic plaque from the surrounding lumen and extravascular tissues. This approach has become well established in the large and relatively immobile carotid arteries, providing detailed information about the presence, burden, and composition of atherosclerotic plaque (24). However, translation of black-blood plaque imaging to the coronary arteries has proved challenging. Indeed, although coronary plaque assessments are possible in individual lesions or arteries (25) (Central Illustration), more complete plaque assessments across the coronary vasculature as a whole remain elusive and the subject of ongoing research (26).

## Adverse Plaque Characteristics

Coronary CT angiography (CTA) is performed using contrast-enhanced imaging (Figure 1) and can offer detailed visualization and characterization of coronary atherosclerotic plaque, with good agreement compared to intravascular ultrasonography (Figure 1) 27, 28. Iodinated contrast is injected through a peripheral cannula, and imaging is timed to ensure peak contrast enhancement of the coronary vessels. Prospective ECG triggering or retrospective ECG gating is used during a single breathhold. Use of 64-multidetector scanners produces images of the heart composed of a number of sections, whereas scanners with more detectors (256 to 320) and a wider z-axis dimension can acquire images of the entire cardiac volume within a single rotation.

**Figure 2:**
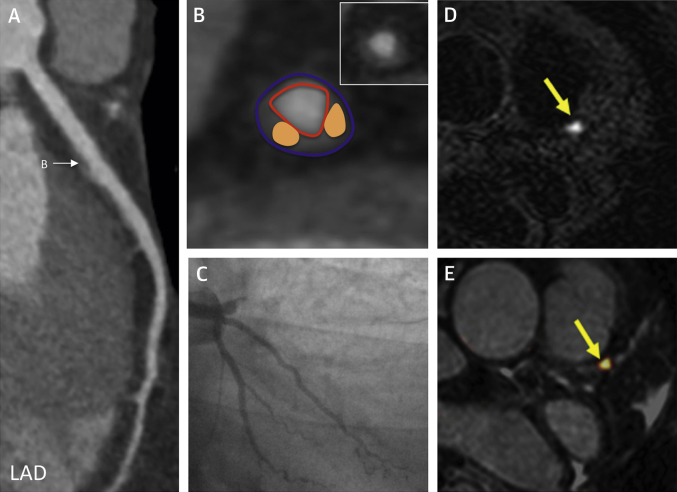
Assessments of High-Risk Plaque Characteristics Coronary CTA shows a nonobstructive plaque in the LAD artery **(A)**. However, this plaque has high-risk characteristics including positive remodeling with a large plaque volume and areas of low attenuation (<30 HU, **orange areas**) on cross-sectional imaging (**image without color** overlay also shown, **inset**) **(B)**. In this patient the plaque in question went on to rupture and cause an acute ST-segment elevation myocardial infarction with occlusion of the LAD **(C)**. Coronary MRA from a different patient demonstrates proximal coronary artery stenosis, which colocalizes with a high-intensity plaque on noncontrast T1-weighted imaging **(yellow arrows) (D, E)**. **D** and **E** adapted with permission from Noguchi et al. (38). LAD = left anterior descending artery; other abbreviations are as in Central Illustration.

Clinically, coronary CTA is widely used to categorize individual plaques as noncalcified (no calcium density), calcified (entire plaque appears as calcium density), or partially calcified (2 visible plaque components, 1 of which is calcified) (29). Beyond this simple categorization, coronary CTA has demonstrated its ability to identify a range of adverse plaque characteristics, including positive remodeling, necrotic core, napkin ring sign, and spotty calcification (Figure 2) (30). Positive remodeling is defined by eccentric plaque formation with relative preservation of the lumen caliber. Regions of low-attenuation plaque (commonly <30 HU) identify a large necrotic core, whereas the napkin ring sign is a CT signature of low attenuation surrounded by a rim of high attenuation. Spotty calcification has been described as a marker of early atherosclerotic calcification (although not true microcalcification) and is also an adverse plaque characteristic (14). Improvements in CT post-processing appear to improve further the delineation of noncalcified plaque, facilitating more accurate assessment of plaque composition (31).

Importantly, several studies have demonstrated that each of these adverse plaque characteristics is more common in patients with ACS than in those with stable angina 14, 32. Moreover, recent data indicate that the prospective identification of these features can identify subjects at increased risk of subsequent ACS. Motoyama et al. (33) studied 3,158 patients with coronary CTA and found high-risk lesions (defined by the presence of both positive remodeling and low-attenuation necrotic cores) in 294 patients who were subsequently 10 times more likely to experience ACS than patients without such plaque (16% event rate in patients with high-risk plaque compared with 1.4% event rate in those without). Similarly, a recent meta-analysis showed that the risk of future ACS was 12-fold higher (odds ratio: 12.1; 95% confidence interval [CI]: 5.2 to 28.1; p < 0.0001) in patients with high-risk lesions than in those with low-risk plaques (30). Although these data support adverse plaque detection as a means of identifying high-risk patients, confirmation in large-scale multicenter clinical trials is required. A current limitation to more widespread use is that the visual assessment of adverse plaque features is both subjective and time consuming; thus, the development of accurate, automated quantification methods is keenly anticipated (34).

The superior soft tissue characterization offered by CMR compared with that of CT is potentially well suited to the detection of adverse plaque characteristics. CMR provides the gold standard characterization of carotid atherosclerotic plaque (24); however, once again, translation of this technology to the coronary arteries has proved challenging. Although the black-blood sequences described earlier can be used to identify positive remodeling (35), only a small proportion of the coronary vasculature can be sampled, and approximately one third of these areas then proves uninterpretable.

CMR approaches aimed at detecting methemoglobin as a marker of acute coronary thrombus are at a more advanced stage of development. Methemoglobin is an intermediate breakdown product of hemoglobin formed 12 to 72 h after hemorrhage or thrombus formation and therefore represents a key component of these acute events. Methemoglobin is associated with a high signal and short T1, allowing fresh thrombi to be detected on CMR in a range of situations, including coronary plaque disruption and intraplaque hemorrhage (Figure 2). In post-MI patients, T1-weighted imaging detected thrombus at the site of the culprit coronary plaque with a sensitivity and specificity of ∼90% (36). In patients with angina, this approach identifies plaque with multiple adverse features on other imaging modalities 37, 38. T1-weighted CMR imaging also appears to provide important prognostic information. In a recent study, high-intensity plaques were observed in 159 of 568 patients with known or suspected coronary artery disease. Forty-one of these subjects subsequently experienced a coronary event, with the presence of a high-intensity plaque acting as an independent predictor on multivariate analysis (hazard ratio [HR]: 3.96; 95% CI: 1.92 to 8.17) (39). A major advantage of this technique compared with other CMR approaches is that these sequences acquire information across the entire volume of the heart and are therefore not limited by sampling error. However, they lack anatomic information, so they must be fused with CMR angiographic scans (Figure 2). Although it is a promising approach, large multicenter studies are required before noncontrast T1-weighted coronary imaging can be recommended clinically.

## Coronary Angiography

Invasive angiography offers unparalleled spatial and temporal resolution. Combined with intravascular ultrasonography and optical coherence tomography, angiography remains the gold-standard for assessing coronary luminal stenoses. It is supported by extensive clinical experience and research data that have confirmed the progressive deterioration in prognosis associated with 1-, 2-, or 3-vessel coronary disease (40). Moreover, it offers the unique opportunity to perform percutaneous coronary intervention immediately after imaging. However, this form of imaging is resource-intensive, invasive, and associated with a small incidence of complications. Additionally, 60% of diagnostic angiograms fail to demonstrate the obstructive coronary disease anticipated (41). The use of noninvasive coronary angiography as a “gatekeeper” to improve patient selection for the catheter laboratory is therefore of major interest.

Coronary CTA demonstrates excellent diagnostic accuracy for the detection of obstructive luminal stenoses compared with invasive coronary angiography. Indeed, a recent meta-analysis described a sensitivity of 93% and specificity of 96% for coronary CTA on a per-segment basis to detect stenoses of >50% (42). Although results in multicenter settings are slightly inferior (sensitivity of 85%, specificity of 90% on a per-vessel basis) (43), they remain sufficient for clinical use. One consistent finding across all trials is the excellent negative predictive value of coronary CTA (>95%) (44), making this technique extremely useful for the exclusion of coronary artery disease. Although rates of nonassessable segments are low (0% to 11%) (45), they are still observed, and commonly relate to motion in the right coronary artery at faster heart rates or partial volume artifact from dense calcification or coronary artery stents (46). It is therefore critical that adequate heart rate control (<65 beats/min) is achieved by using beta-blockade administration. Advancements in scanner hardware and software, including faster scanners, wider detector arrays, increased spatial resolution, new reconstruction algorithms, motion correction algorithms, and dual-source imaging, may further reduce these artifacts (47). Although coronary CTA is, by necessity, associated with radiation exposure, this has been reduced substantially and is now routinely lower than nuclear imaging modalities. For example, in SCOT-HEART, the median radiation dose for CT imaging, including coronary CTA and CAC scoring, was 4.1 (3.0 to 5.6 mSv) (48).

Coronary CTA assessments of coronary artery stenosis provide important prognostic information. Similar to coronary angiography, a stepwise deterioration in prognosis associated with 1-, 2-, and 3-vessel disease has been observed using coronary CTA (49). Coronary CTA has also documented the adverse prognosis associated with nonobstructive disease (not detected with standard perfusion assessments) and confirmed the excellent prognosis for patients with a normal coronary CTA, with a risk of cardiovascular events of <0.5% that extends up to 5 years (50). Coupled with the excellent negative predictive value of a normal scan, coronary CTA is recommended in ruling out coronary artery disease in those considered at low or intermediate risk (51).

Recently, 2 large multicenter randomized controlled trials (RCTs) have assessed the utility of outpatient coronary CTA in patients with stable chest pain. The PROMISE (PROspective Multicenter Imaging Study for Evaluation of chest pain) trial randomized 10,003 symptomatic stable outpatients to initial evaluation with coronary CTA or to functional testing (52). Patients were a mean 62 years of age, 53% were female, and the pre-test probability of obstructive coronary artery disease was 53%. There were no differences in outcomes between the 2 strategies at 25 months follow-up, indicating that coronary CTA can provide effective risk stratification and be safely used in the management of outpatients with suspected coronary heart disease. The SCOT-HEART study used a different strategy, randomizing 4,146 outpatients with suspected angina due to coronary heart disease to standard care (including high rates of stress testing) or standard care plus coronary CTA. Patients were a mean 58 years of age, 44% were female, and the pre-test probability of obstructive congenital heart disease was 47%. The addition of coronary CTA provided improved diagnostic certainty and reduced rates of normal coronary angiography. Post hoc analysis showed that coronary CTA led to more appropriate use of revascularization and preventive therapies associated with a halving of fatal and nonfatal MI 48, 53. Reduced MI rates among stable patients assessed with coronary CTA versus standard care were also observed in a recent meta-analysis including >14,000 patients (54).

Several RCTs have also assessed the clinical utility of coronary CTA in patients who attend the emergency department with acute chest pain (CT-STAT [Coronary Computed Tomographic Angiography for Systematic Triage of Acute Chest Pain Patients to Treatment], ROMICAT-II [Rule Out Myocardial Ischemia/Infarction Using Computer Assisted Tomography], ACRIN-PA [CT Angiography for Safe Discharge of Patients with Possible Acute Coronary Syndromes]) 55, 56, 57, most of whom do not have ACS. These studies have demonstrated that early coronary CTA accelerates diagnosis and, as a consequence, expedites either discharge or initiation of therapy. A meta-analysis showed that coronary CTA reduced hospital stay and expenditure, while increasing rates of invasive coronary angiography and revascularization (58). On the basis of those studies, the use of coronary CTA in the assessment of patients with stable angina and in the emergency department has now entered routine clinical practice.

CMR angiography offers major technical challenges and, to date, has lagged behind coronary CTA. However, CMR angiography potentially holds several key advantages over CT, maintaining interest in this method (Figure 1) (26). First, CMR angiography is not affected by the calcium-related artifacts that can limit the utility of coronary CTA imaging in patients with advanced atheroma. Second, and perhaps most importantly, CMR angiography does not involve exposure to ionizing radiation. This is particularly attractive with respect to screening programs for the imaging of younger patients with earlier stage disease and for serial imaging to track disease progression over time. Indeed, it is worth noting that CMR, not CT, has emerged as the imaging modality of choice for assessing the carotid and peripheral arteries, with intense research focused on translating these same techniques to the coronary arteries.

It takes much longer to acquire CMR angiography data than CTA data, so imaging within a single breath-hold is not currently feasible. As a consequence, the major challenge facing CMR angiography is how to successfully correct for coronary arterial motion while acquiring sufficient information for high spatial resolution (26). Current sequences rely upon ECG gating coupled with a respiratory navigator so that CMR data are acquired only in diastole, when the diaphragm is close to its resting position. This approach is highly wasteful, with most of the data being ignored. Scans are therefore time consuming, and even the fastest CMR angiography sequences take ∼10 min compared with the seconds or even milliseconds required for coronary CTA. Moreover, the metallic artifact that arises from intracoronary stents on CMR remain unresolved. Nevertheless, CMR angiography allows assessment of the proximal and midcoronary vessels with an accuracy approaching that of CTA (26). Kim et al. reported the first multicenter study to investigate the accuracy of CMR angiography compared to invasive angiography, demonstrating a sensitivity of 93% but a disappointing specificity of 42% for obstructive coronary artery disease (59). A subsequent meta-analysis in 2010 showed improvement, with an overall sensitivity of 87% and a specificity of 70% (60). With further technological advances, the latest techniques (noncontrast, free-breathing, 3-dimensional whole-heart steady-state free precession scans) report further improvements in accuracy (sensitivity of 91%, specificity of 86%, area under the curve of 0.92), although this remains inferior to coronary CTA (61). Consequently, the most recent Appropriateness Use Criteria do not recommend the use of CMR angiography for the assessment of native coronary arteries (62). However, 2 areas where CMR angiography is recommended are in the assessment of anomalous coronary arteries with aberrant origins (Figure 1) and in the detection of coronary aneurysms. CMR angiography can provide accurate visualization of these proximal, large-caliber abnormalities and avoid radiation exposure in young patients, who are the most commonly affected.

## Flow Obstruction and Myocardial Perfusion

Stenoses of the coronary vasculature can limit blood flow to the myocardium, resulting in ischemia and anginal symptoms during periods of increased demand (e.g., exercise). However, the degree of coronary stenosis and the extent of myocardial ischemia do not correlate well; thus, noninvasive assessments of flow obstruction and myocardial perfusion retain an important role in patients with these symptoms.

**Figure 3:**
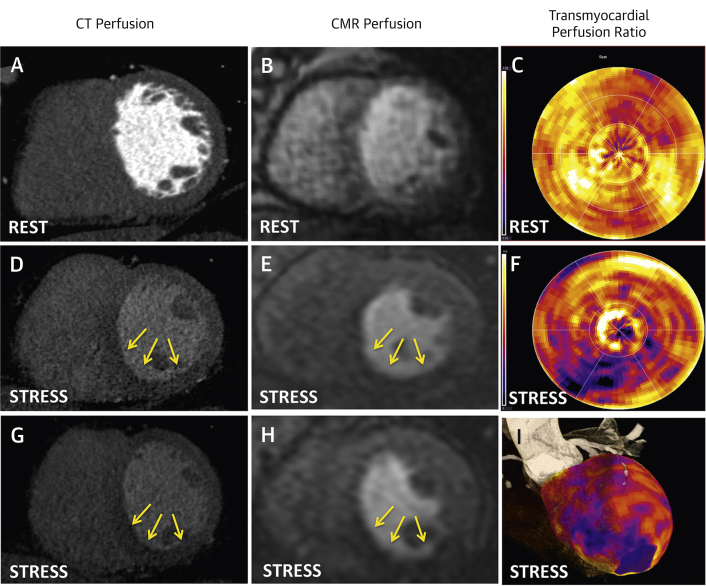
Myocardial Perfusion Imaging Myocardial perfusion imaging in a single patient using both CMR and CT with an adenosine pharmacological stress protocol. Normal left ventricular wall perfusion can be observed at rest using CT **(A)** and CMR **(B)** to generate a map of the transmyocardial perfusion ratio **(C)**. Under stress conditions, perfusion defects **(yellow arrows)** in the basal inferior and inferoseptal walls on CT **(D),** CMR **(E),** and the transmyocardial perfusion ratio map **(F),** with defect also seen at midcavity level **(G, H)**. Combined 3-dimensional coronary CTA and CT myocardial perfusion image **(I)**. Abbreviations as in Central Illustration.

CT can provide functional assessments of coronary flow obstruction by using 2 major approaches: myocardial perfusion imaging and noninvasive CT-derived fractional flow reserve (FFR_CT_). CT myocardial perfusion imaging involves assessment of the passage of iodinated contrast medium into the myocardium during vasodilator stress (e.g., adenosine, dypyridamole, or regadenoson) (Figure 3). Two different protocols have been established, namely, the “snapshot” and “dynamic” techniques. The “snapshot” protocol involves acquisition of 1 image (or a small number of images) at the peak of myocardial enhancement, limiting radiation exposure. Perfusion defects are identified as a comparison between areas of reduced myocardial attenuation density and normal or resting myocardium (Figure 3). The “dynamic” protocol obtains multiple images during both contrast wash-in and wash-out. This is similar to CMR or positron-emission tomography perfusion imaging, allowing calculation of myocardial blood flow. The largest multicenter study of CT perfusion imaging to date, the CORE320 (Computed tomography angiography and perfusion to assess coronary artery stenosis causing perfusion defects by single photon emission computed tomography) study, used the snapshot approach and demonstrated a sensitivity of 80% and specificity of 74% for the presence of obstructive coronary artery disease compared to single-photon emission computed tomography (SPECT) (63). A recent meta-analysis identified a pooled sensitivity of 75% to 84% and a specificity of 78% to 95%, depending on the protocol used (64). Although CT perfusion involves additional radiation exposure, it may find a useful role as an adjunct to coronary CTA in the presence of indeterminate stenoses.

Computational flow dynamics have been used to calculate FFR from coronary CTA images (FFR_CT_). Several multicenter studies have compared FFR_CT_ with FFR assessed during invasive angiography (e.g., the DISCOVER-FLOW [Diagnosis of Ischemia-Causing Stenoses Obtained Via Noninvasive Fractional Flow Reserve] and DeFACTO [Determination of Fractional Flow Reserve by Anatomic Computed Tomographic Angiography] studies) 65, 66. A recent meta-analysis found that FFR_CT_ had a pooled sensitivity similar to coronary CTA (0.83 vs. 0.86, respectively, per-vessel analysis) but improved specificity (0.78 vs. 0.56, respectively, per-vessel analysis) (67). Recently, the PLATFORM (Prospective LongitudinAl Trial of FFRct: Outcome and Resource IMpacts) study showed that FFR_CT_ reduced the number of subsequently normal invasive coronary angiograms compared with standard care, reducing costs and improving quality of life (68). However, this study did not directly compare FFR_CT_ with the results of coronary CTA alone, so the incremental benefits of FFR_CT_ over those of CTA were not studied. Moreover, this form of image analysis is expensive, and it remains unclear how it deals with the problems of calcium artifacts and cardiac motion that often underlie indeterminate lesions.

CMR can detect myocardial ischemia by assessing both myocardial perfusion and changes in left ventricular wall motion in response to stress. CMR perfusion is most commonly performed at rest and peak vasodilator stress following the bolus administration of gadolinium. On first pass perfusion regions of myocardial ischemia are relatively hypoperfused, resulting in a reduced or delayed peak in the myocardial signal intensity (Figure 3). Reversible defects can then be differentiated from areas of MI, identified on rest perfusion or late gadolinium enhancement (LGE). CMR perfusion has been tested in several large clinical trials, demonstrating high diagnostic accuracy (69) that was at least equivalent to that of SPECT perfusion imaging in the recent CE-MARC (Cardiovascular magnetic resonance and single-photon emission computed tomography for diagnosis of coronary heart disease) clinical trial (70). This trial also recently demonstrated that CMR perfusion provides prognostic information that is improved compared with that of SPECT (71). A recent large meta-analysis demonstrated a pooled sensitivity of 89% and a specificity of 78% for the detection of obstructive coronary artery disease (72) and that a negative CMR stress perfusion test was associated with an excellent prognosis (73). Although SPECT is much more widely available in the United States and supported by strong prognostic observational data, CMR perfusion has the crucial advantage of being radiation-free and appears to be cost effective (74). The 2013 European Society of Cardiology guidelines on the management of stable coronary artery disease recommend stress perfusion CMR as 1 potential imaging option for the assessment of patients presenting with chest pain and a pre-test probability of coronary artery disease of 15% to 85% (Class I recommendation; Level of Evidence: B) (51). CMR perfusion appears to be particularly clinically useful in patients with severe coronary artery disease when additional information related to myocardial viability and myocardial function can aid decision making. CMR perfusion therefore potentially complements coronary CTA, which, as discussed, is best used at the milder end of the disease spectrum.

Similar to stress echocardiography, CMR can detect obstructive coronary artery disease based upon the detection of wall motion abnormalities that develop in response to low-dose dobutamine stress (75). CMR perfusion imaging is usually preferred, perhaps due to concerns regarding inotrope administration in the scanner, although major complications appear to be rare (75).

## Imaging Myocardial Infarction, Viability, and Function

The presence of persistent contrast on delayed imaging can be used to identify scarring or fibrosis in the left ventricular myocardium, using CMR and CT. Both iodinated CT contrast agents and gadolinium wash out of regions of replacement myocardial fibrosis or scarring more slowly than areas of normal healthy myocardium. Increased signal/attenuation are therefore observed in these areas if imaging is performed at delayed time points, informing about both the presence and pattern of myocardial scarring in the left ventricle.

**Figure 4:**
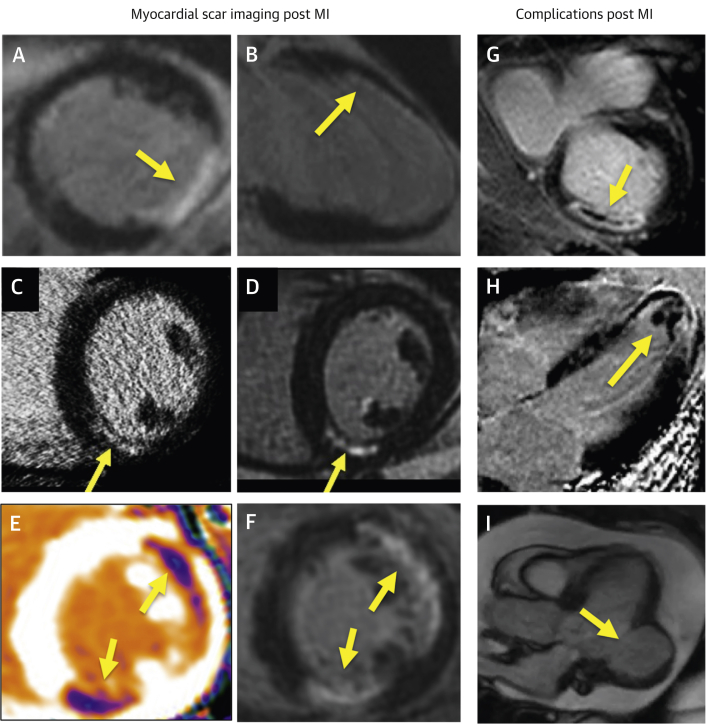
Assessments of Myocardial Scar, Viability, and Complications Post Infarction CMR LGE techniques can visualize both transmural **(A)** and subendocardial **(B)** myocardial infarction. Infarct visualization is also possible with CT late contrast enhancement **(C)** versus CMR LGE in the same patient **(D),** and CMR T1 mapping **(E)** versus LGE in the same patient **(F)**. Complications following myocardial infarction, such as microvascular obstruction **(G)**, apical thrombus **(H)**, and pseudoaneurysm **(I)** can be readily seen on CMR to help guide further management. **Yellow arrows** point to each imaging feature mentioned in each part. LGE = late gadolinium enhancement; other abbreviations as in Central Illustration.

The first description of MI detection using late enhancement was, in fact, made with CT in 1976 (76). More recently, CT late enhancement has demonstrated moderate diagnostic accuracy in the detection of MI (sensitivity of 52% to 78%, specificity of 88% to 100%) (77), with several techniques (e.g., low tube voltage, dual-energy imaging, and increased contrast volume) being explored to improve scar visualization (78) (Figure 4). However, the inferior image quality and associated radiation exposure (1 to 5 mSv) mean that CMR late enhancement techniques are currently preferred.

Over the last 2 decades, LGE CMR has become the gold standard method for detecting myocardial scar. Indeed, the principal strength of CMR has been the detailed myocardial tissue characterization it provides. Different pathologies demonstrate different patterns of scarring and, consequently, different patterns of LGE. For example, previous MI is associated with subendocardial LGE (the myocardial region farthest from the epicardial coronary arterial system) that extends transmurally as vessel occlusion persists (Figure 4). This can be clearly differentiated from the linear mid-wall pattern (or absence of LGE) observed in dilated cardiomyopathy, helping clinically to differentiate between these 2 pathologies (79). Similarly, in patients presenting with acute troponin-positive chest pain and nonobstructive coronary angiography, CMR can help differentiate between MI and other potential diagnoses such as myocarditis or Takotsubo cardiomyopathy (80).

Importantly, regardless of the pattern of fibrosis or the cardiac condition, the presence of myocardial LGE is consistently associated with an adverse prognosis 81, 82. Following MI, multiple large studies have demonstrated that infarct size measured by CMR LGE is a stronger predictor of outcome than either left ventricular volumes or ejection fraction 83, 84.

The detection of prior MI using LGE also appears more sensitive than previous techniques (e.g., ECG analysis and nuclear techniques), allowing us to appreciate the relatively high prevalence of unrecognized MI in at-risk populations. Kwong et al. (85) studied 195 patients (mean 59 ± 13 years of age, 68% male) with symptoms or signs suspicious for ischemic heart disease but without a previous diagnosis of MI. They observed subendocardial MIs in 23% of their patients (n = 44), often involving only a small area of the myocardium (85). In the ICELAND-MI (Prevalence and Prognosis of Unrecognized Myocardial Infarction Determined by Cardiac Magnetic Resonance in Older Adults) study (86), 936 patients (mean 76 years of age, 48% male) underwent CMR, with 10% (n = 91) found to have previously unrecognized MI. In a subgroup of 377 diabetic patients, previously unrecognized MI was observed in more than 20% (n = 72) (86). Importantly, in each of these studies and others, the presence of previously unrecognized MI was a strong independent predictor of prognosis 85, 86, 87.

Aside from its prognostic utility, CMR LGE is increasingly being used clinically to assess myocardial viability. In 2000, Kim et al. (88) assessed the ability of LGE to predict functional recovery in hypokinetic areas of myocardium following revascularization (n = 50 patients and 804 hypokinetic segments) (Figure 4). In segments with ≤25% transmurality of scar, ∼80% recovered function, whereas only 10% of segments recovered when the transmurality was >50% (88). This has been confirmed in several other small, single-center studies 89, 90. A remaining difficulty is the segments with 25% to 50% transmurality, which demonstrate a roughly 50:50 chance of functional recovery, although in these cases, addition of a low-dose dobutamine study can help improve diagnostic accuracy (84). Measuring the thickness of viable tissue around the rim of the infarct provides an alternative assessment of viability, with measurements >4 mm associated with an increased likelihood of functional recovery 91, 92. In contrast, myocardial wall thickness is not necessarily a reliable guide. Shah et al. (93) recently demonstrated that 18% of thinned (<5.5 mm) akinetic myocardial segments have evidence of only limited LGE (<50% transmurality) and that these regions frequently demonstrated improved contractility and resolution of thinning following revascularization (93). Despite the supportive observational data and the expanding clinical experience with LGE in assessing viability, definitive evidence demonstrating improved clinical outcome following CMR-guided revascularization remains lacking. This is urgently required, particularly given the failure of alternative viability assessments to improve prognosis in the STITCH (Surgical Treatment for Ischemic Heart Failure) trial (94).

CMR can provide myocardial tissue characterization beyond the presence of myocardial scar, with the ability to identify myocardial necrosis, edema, hemorrhage, microvascular obstruction, and left ventricular thrombus. This is of use in the early stages following acute MI, when scar has yet to develop but where LGE uptake still occurs in regions of myocardial necrosis and edema. Indeed, in these early stages following MI, LGE tends to overestimate the size of the scar that will ultimately develop. LGE imaging can also be used to detect microvascular obstruction. This appears as a dark core within the otherwise bright regions of late enhancement and is a feature of large transmural infarcts correlating with the angiographic no-reflow phenomenon and myocardial hemorrhage (Figure 4). In addition to infarct size, it heralds a poor prognosis for patients post MI of incremental value to standard predictors (84).

Other sequences can also be used to visualize acute MI. T2-weighted short-tau inversion recovery (STIR) imaging detects regions of myocardial edema, helping to differentiate acute from chronic infarction and to allow calculation of the myocardial area at risk (95). Novel T1 mapping techniques can go beyond LGE imaging to identify more diffuse, interstitial forms of fibrosis in the remote myocardium and peri-infarct area (96). Finally, early imaging, 1 to 4 min following gadolinium injection, allows detection of intracardiac thrombi with improved sensitivity (88%) compared with echocardiographic techniques 97, 98 (Figure 4).

The assessment of global and regional function in the left ventricle is performed routinely using echocardiography. This provides important prognostic and diagnostic information in the assessment of patients with ischemic heart disease and is used to guide implantation of automatic implantable cardioverter-defibrillators and cardiac resynchronization therapy. In patients with poor echocardiographic windows or in cases of diagnostic uncertainty, CMR can be used to assess left ventricular function. Indeed, CMR is widely considered the noninvasive gold standard for these measurements (62). Similar assessments are also available with coronary CTA using images acquired throughout the cardiac cycle, although this again involves radiation exposure (3 to 10 mSv) and is rarely performed in clinical practice.

## Barriers to Widespread Clinical Adoption

CT and CMR offer detailed and comprehensive imaging of patients with ischemic heart disease. The prognostic implications of these assessments are now well established, with emerging studies demonstrating the ability of these modalities to change patient management and improve clinical outcomes, such as cardiac events and mortality. However, several barriers to their widespread clinical adoption remain. CT imaging will need to continue to reduce radiation doses, particularly in the context of perfusion, viability, and functional assessments. The use of CT in the emergency department will require rapid access to scanners and the availability of trained technologists and reporters. For patients with stable chest pain, there is currently a wide choice of noninvasive imaging modalities, with further work required in order to determine how best to incorporate CT within the patient care pathway. The development of noncontrast CMR approaches, faster imaging protocols, and CMR-compatible permanent pacemakers and automatic implantable cardioverter-defibrillators will also be important in increasing the clinical utility of this modality. Currently, CMR is a time-consuming process with respect to both image acquisition and interpretation. However, improvements in software, hardware, and, in particular, motion correction (removing the need for ECG gating) appear set to greatly improve the efficiency and accessibility of CMR. Concerns also exist about administration of gadolinium-based contrast medium. These agents are widely used in radiological practice, with overall an excellent safety profile (the incidence of acute adverse reactions is 0.1% to 0.2%). However, they should be avoided, if possible, in patients with advanced renal dysfunction (glomerular filtration rate <30 ml/min/1.73 m^2^), in whom the rare but serious complication of nephrogenic systemic fibrosis has been reported (99). Moreover, the accumulation of gadolinium in the brain after repeated administration has recently been described, even in patients with normal renal function (100). This led the U.S. Food and Drug Administration to issue a drug safety communication (101), although the clinical implications of this observation remain unclear.

Perhaps the major barrier to the widespread clinical adoption of cardiac CT and CMR is the relative expense and limited availability of these techniques. This is especially the case in the developing world, where the burden of ischemic heart disease is rising rapidly. However, even in the developed world, modalities such as ultrasonography and nuclear imaging are more accessible and can be performed at reduced cost. In the current era of constrained healthcare expenditure, advanced imaging techniques, such as CT and CMR, will therefore need to find ways to complement these existing approaches and to demonstrate not only their clinical superiority, but also their cost effectiveness.

## Conclusions

CT and CMR imaging of the coronary arteries and left ventricular myocardium provides complementary and comprehensive assessments of ischemic heart disease from its earliest pre-clinical stages to the final phases of advanced cardiac failure. These novel imaging modalities are already affecting clinical care, with further advances set to expand their utility and role. In particular, CT has emerged as the noninvasive test of choice for imaging the coronary vasculature, demonstrating clinical efficacy in multiple large-scale RCTs. CMR, by contrast, is of major value in assessing the left ventricular myocardium, in particular, its ability to investigate myocardial perfusion and tissue composition without the need for ionizing radiation. With ongoing and rapid technological development, the clinical utility of both approaches is set to expand still further, although their widespread adoption will require further RCTs demonstrating improved clinical outcomes and cost-effectiveness.
